# COVID-19 Vaccination in Pediatric Population: A Necessity or Obstruction to the Protection of the Right to Health? Biojuridical Perspective

**DOI:** 10.3389/fpubh.2022.874687

**Published:** 2022-05-30

**Authors:** Clio Bilotta, Giulio Perrone, Stefania Zerbo, Antonina Argo

**Affiliations:** Department of Health Promotion, Mother and Child Care, Internal Medicine and Medical Specialties, Section of Legal Medicine, University of Palermo, Palermo, Italy

**Keywords:** COVID-19 vaccination in pediatric population: bioethical controversy COVID-19 vaccination, pediatric population, children, right to health, bioethical controversy

## Abstract

One of the most recently debated topics worldwide is the mass vaccination of children against coronavirus disease 2019 (COVID-19). Next, the risk/benefit ratio of COVID-19 vaccination and infection in children are compared. Nonetheless, the real question in this debate is as follows: Does the vaccine represent a necessary tool or is it an obstacle in protecting the right to health? From a public health point of view, the Supreme Court of Nova Scotia, in Canada, recommends COVID-19 vaccination in the pediatric population. Based on Article 25 of the Draft Articles on State responsibility, vaccination can be considered a social act necessary for protecting the individual's right to health. The 1989 New York Convention on the Rights of the Child and the European Regulation number 219/1111 state that the opinion of a minor aged >12 years is considerable. However, this validity of opinion is related to age and degree of discernment. The onset of adverse events following the administration of the COVID-19 vaccine may lead to compensation in the near future. Recent studies have identified a new COVID-19-related pediatric pathology, known as multisystem inflammatory syndrome. Other studies have demonstrated that myocarditis in the pediatric population might occur following COVID-19 vaccine administration. In June 2021 in the USA, the Center for Control and Prevention of Infectious Diseases Advisory Committee on Immunization Practices declared that the benefits of vaccination against COVID-19 in the pediatric population outweighed the risks. In the meantime, whereas the bioethical debate remains open, monitoring the real risk/benefit ratio of vaccination in the pediatric population is crucial.

## Introduction

The ongoing coronavirus disease 2019 (COVID-19) pandemic caused by severe acute respiratory syndrome coronavirus-2 (SARS-CoV-2), which first began in 2020, led several countries worldwide to adopt drastic preventive measures to reduce infection rates and mortality. The pandemic led to profound changes. Most individuals were subjected to restrictive measures that affected their freedom, to protect their health, which is considered a legally protected right. Various debates are ongoing regarding the legitimacy of the preventive measures taken during the COVID-19 pandemic and their concordance with the protection of human rights. In fact, the content of several decree laws, such as Decree Law No 28/2020 and Decree Law No 6/2020, created a dichotomy between the right to personal freedom and the right to health.

In the wake of the COVID-19 pandemic, one of the most recently debated topics worldwide concerns the mass vaccination of children against COVID-19. It is certainly not the first time that the literature addresses the thorny issue regarding the dichotomy between individual freedom and public health, in the case of massive and/or mandatory vaccinations. The erroneous connection between the measles-mumps-rubella vaccine and autism, which was reported by Andrew Wakefield et al. in 1998, despite the subsequent official denials (1, 2), contributed to the no-vax movement, decreasing vaccination rates (3, 4), and resurgence of vaccine-preventable diseases (5).

The spotlights on the mass vaccination of children against COVID-19 are relatively recent, since the vaccination campaign first involved elderly people. In fact, as it is now well-known, the lethality of COVID-19 is significantly higher in older individuals. Around 80% of COVID-19-related deaths in China predominantly involved individuals aged >60 (6), and the highest mortality rate was recorded in Europe in individuals aged ≥75 years (7, 8). Consequently, several questions arose regarding COVID-19 vaccination in the pediatric population. In fact, a debate was present regarding the age groups to be vaccinated. When the Food & Drug Administration in America authorized mRNA vaccines in the pediatric population aged ≥12 years, both the American Academy of Pediatrics and the Center for Control and Prevention of Infectious Diseases recommended vaccination in this age group. On the other hand, Great Britain recommended vaccination in children aged 12–17 years, only in cases of severe chronic disease, or if they were living with vulnerable individuals. The Public Health Agency maintained a prudent approach by watching the impact of vaccination in other countries such as Sweden. Most recently, both the Food & Drug Administration and European Medicines Agency authorized the use of mRNA vaccines in the 5–11-year age group. The discussion that followed was regarding the comparison of the risk/benefit ratio of COVID-19 vaccination and the risk of infection in children.

Previous epidemiological data, which drive the current opinion that is adverse to the administration of COVID-19 vaccines to pediatric population, showed an asymptomatic or paucisymptomatic course of SARS-COV-2 infection in children and adolescents (9, 10), with a hospital admission rate of <2% (11) and a low mortality (12). Nonetheless, subsequent studies demonstrated that the initial data underestimated COVID-19-related pediatric hospitalizations and mortality (13). These studies noted that the hospital admission rate was 11.7%, with severe manifestations of infection in 3.6% of cases (14). Over 5.7 million pediatric cases of SARS-CoV-2 were diagnosed in 2021 in the USA, with 21,814 hospitalizations and 498 deaths (9).

While monitoring 15 hospitals, the Association of Italian Pediatric Hospitals pointed out that in January 2022, pediatric patients' admission rate to the intensive care unit and/or any other medical floor, due to COVID-19 infection, was much greater than in the three previous pandemic waves^1^.

The Italian Society of Pediatrics encouraged pediatric COVID-19 vaccinations. They argued that, although symptoms from COVID-19 infection in children are commonly milder than in the adult population, several cases of severe infection required urgent hospitalization in the intensive care unit, and deaths occurred in the pediatric population. For this reason, the Italian Society of Pediatrics raised the issue of protecting children's health by administering the vaccine. This gave the pediatric population the same rights as the adult population. The vaccine was therefore considered a necessary element in protecting the health of the pediatric population ^2^.

However, all medication, including the COVID-19 vaccine (15), are not exempt from adverse effects. In fact, they sometimes carry even serious and/or lethal consequences. Therefore, the question that arises is that “is it really necessary to administer a vaccine, with unknown side effects, in a young population, in which the incidence of COVID-19-related complications and deaths seem low?” However, when considering this argument, it is necessary to evaluate the late complications of COVID-19 infection, to be able to compare them with those of the vaccine. It is clear that the answer to these questions is inherent in the evaluation of the risk/benefit ratio, which could currently be distorted by early data from inconclusive studies.

Therefore, similar to the adult population, a pertinent question still weighs on this very complex issue: Does the vaccine represent a necessary tool to protect the right to health of children and the global population, or rather an obstacle against the protection of the same right?

## Multifactoriality of the Right to Health

One of the aspects of each vaccination campaign is protecting the right to health of the global population and the individual himself. The first two international sources that included the right to health among other human rights were the Universal Declaration of Human Rights (The Universal Declaration of Human Rights – United Nations General Assembly of 10 December 1948), which mentioned health as an essential element for an adequate standard of living (Article 25) and the WHO Constitution (Constitution of the World Health Organization – 7 April 1948), which declared that health is “a state of complete physical, mental and social well-being, and not merely the absence of disease or infirmity.” This right was further recognized as a human right in the International Covenant on Economic Rights Social and Cultural events in 1966 (International Covenant on Economic, Social, and Cultural Rights – General Assembly of 16 December 1966).

Many subsequent conventions, such as the European Social Charter, the American Convention on Human Rights, and the International Convention on Economic, Social, and Cultural Rights, contain an article or a clause relating to the right to health. The International Convention on Economic, Social, and Cultural Rights established in article 12 that states should “recognize the right of everyone to the enjoyment of the highest attainable standard of physical and mental health” that they are able to achieve. Regarding minors, paragraph 1 of article 24 of the International Convention on the Rights of the Child and Adolescent reports that the states recognize the right of the minor to secure the best possible state of health and to benefit from medical services and rehabilitation. This convention strives to ensure that no minor is deprived of the right to have access to such health-related services.

## Comparison of Case-Law Trend About Pediatric Vaccination

If the jurisprudence is already favorable to mandatory vaccination, despite scientifically proven adverse effects secondary to vaccines' administration, it is understandable how the same jurisprudence could be in favor of the vaccination campaign against COVID-19 in the pediatric population. In fact, recent judgments in Italy link vaccination to the aforementioned concept of health, which is understood as a set of socioeconomic benefits, as well as health, establishing that the non-fulfillment of the vaccination obligation constitutes an obstative reason, in itself, to access the schools of the childhood (pursuant to art.3 paragraph three of Legislative Decree no. 73/2017), to protect the minors and the entire school community (Regional Administrative Court of Piedmont, judgment no. 1034/2018). Comparative jurisprudence deals with the same theme: in England, the Family Division (Lincoln) of the High Court of Justice, sided in favor of the administration of mandatory vaccines, going against the doctrine that the potential risks of vaccines are greater than their benefits in children. The uncertainty regarding the long-term side effects was not given much importance. These currents of thought would be based, according to the judges, on general public knowledge, rather than on academic scientific knowledge.

The same judgment was issued by the Family Division of the Supreme Court of Nova Scotia, in Canada, which, in the decision reasons, highlights the positions of the Public Health Agency of Canada and the World Organization of Health, recommending the vaccinations in support of public health and arguing that contrary opinions are not supported by any scientific evidence and, therefore, not suitable for the protection of the best interest of the minor.

## The State of Necessity: A Jurisprudential Exemption?

In the case of the COVID-19 pandemic, the concept of necessity is relevant. The need to counter the spread of the COVID-19 pandemic within one's own territory can erase the unlawfulness of certain conducts, opposing international laws. Article 25 of the Draft Articles on State Responsibility of the International Law Commission (2001) discusses the following state of necessity to commit the deed to avoid a serious, imminent, and involuntary danger, which is not punishable. The proclamation of a state of emergency in 2020 led to a succession of numerous sources of law, differing in strength and effectiveness. Examples of such laws are the resolutions of the Council of Ministers, decree laws, ministerial decrees and Prime Ministerial decrees, ministerial orders, civil protection orders, and trade union ordinances. The questions that arise are the relationship between these sources and the Constitution, and whether it is legitimate to apply these non-legislative sources to a fundamental constitutional right (16). The Constitutional Court specified that such acts must respect the general principles of the legal order and constitutional rights and that they must be in accordance with the state of emergency. Failure to comply with these conditions would determine the constitutional illegitimacy of these measures.

## The Informed Consent of the Minor

The Italian legislation states that the consent of a single parent is sufficient when it comes to ordinary acts involving a minor. In contrast, the consent of both parents is required for extraordinary acts. Based on these legislations, routine treatments, such as, mandatory medications and vaccinations represent ordinary acts. All other medical acts are classified as extraordinary. Vaccination against COVID-19 constitutes an extraordinary act, since it is not yet compulsory. As such, consent from both parents is required, and a mandatory double signature is requested in a filled format provided by the National Health Ministry.

In the event of a conflict between the parents, or between them and the minor, the conflict is resolved by the judge of the Juvenile Court. In the case of disagreement of the minor, who is “capable of discernment,” a large proportion of jurists considers that a health treatment contrary to the minor's will infringes the principle enshrined in article 32 of the Constitution.

In fact, the 1989 New York Convention on the Rights of the Child, the 1997 Strasbourg Convention on the Rights of the Child, the European Constitution, and the European Regulation no. 219/1111 expressly state that the opinion of the minor who aged >12 years has its weight. However, the age of the minor and his degree of discernment are taken into consideration (17). Medico-legal doctrine addressed this topic while having a similar position, taking into account the opinion of minors aged ≥14 years, when it comes to the topic of preventable infectious diseases (18).

## COVID-19 Vaccination: Future Prospects of Compensation?

The onset of any acute adverse event following COVID-19 vaccination may lead to consequences in the near future. In fact, constitutional judges are based their reasoning on the existence of the duty of social solidarity with those who have to undergo a certain act of prophylaxis for the protection of the global health. In this regard, the Italian sentence no. 107 of the Constitutional Court, which took place on 26 April 2012, declared the unconstitutionality of article 1, paragraph 1, Lex 25 February 1992, no. 210. This was done because the latter article does not provide the right to compensation, under the conditions and in the manners established by the same law, in respect of those who have suffered consequences following vaccination against measles, mumps, and rubella.

## Comparison of Risk/Benefit Ratio of COVID-19 Infection and Vaccine

Although with a rather low incidence, COVID-19 still can cause complications in the pediatric population. In fact, recent studies have identified a new entity, known as multisystem inflammatory syndrome (MIS-C), or pediatric inflammatory multisystem syndrome (PIMS-TS). This syndrome was considered a sequelae of COVID-19 based on the very short time interval between the infection and the onset of this syndrome. In some cases (3–25%), patients had comorbidities, such as immunological diseases, immunosuppression, cancers, and respiratory diseases. Severe complications of this syndrome include respiratory and myocardial failure (19). Other complications of COVID-19, commonly in the adult population, concern the onset of long-term effects, up to 6 months after infection, consisting of multiple symptoms, including fatigue, muscle, bone and joint pains, palpitations, insomnia, and breathing problems (20). These findings are also seen in the pediatric and adolescent population.

Although the vast majority of subjects in the pediatric and adolescent population did not report complications following COVID-19, both MIS-C and prolonged COVID-19 could have permanent effects, especially for patients with comorbidities, influencing their future development and health (21, 22). As such, vaccination is crucial to prevent these negative outcomes.

Vaccination would also allow social reintegration (23), further lowering the rate of spread of the virus within the population. Nevertheless, some studies demonstrate the presence of adverse reactions following COVID-19 vaccination, such as the onset of myocarditis (24). The incidence rate can be high, especially in males aged 12–29 years (25). Nevertheless, all myocarditis cases resolved shortly after occurrence.

As such, in June 2021 in the USA, the Center for Control and Prevention of Infectious Diseases Advisory Committee on Immunization Practices declared that the benefits of vaccination against COVID-19 for the pediatric population outweighed the risks and therefore recommended vaccination in subjects aged ≥12 years (26).

Finally, a point worthy of consideration in this emerging ethical debate is the position of some authors who highlighted the presence of masqueraded childism while deprioritizing children vaccination in the face of a vulnerable adult population (27). The arguments for a better natural immunity generated through infection, and the possible risk of vaccination in children, seem to be related to this unequal moral approach. Therefore, to fight hidden childism, more efforts should be put into combatting injustice against children's health (28, 29).

## Comparison With Others Vaccines: Judicial Aspects

In 2017, in an attempt to counter the outbreak of epidemics caused by lower vaccination rates in the pediatric population, the Italian Parliament extended the list of mandatory vaccinations to include those against pertussis, measles-mumps-rubella, chickenpox, and Hemophilus influenzae type B (30, 31). In fact, pediatric vaccination rates had reached percentages much lower than the 95% coverage target vaccination rate (32). Mandatory vaccination resulted in the prohibition of access to pre-school education services for children aged <6 years, if they were not vaccinated. Although this law allowed an increase in the rate of vaccination, the idea of mandatory vaccination remains a controversial and debated issue (33). Prior to the introduction of this new law, the communication campaigns were completely inadequate (34). Meanwhile, France also made some pediatric vaccinations mandatory to reduce the increased mortality secondary to decreased vaccination rates (35). Similar legislative measures have been adopted by California's government in 2015, forcing the country to pass Senate Bill 277/2015, a law that removes non-medical exemption from school vaccinations (36). Although measles vaccination coverage increased to over 95% since the introduction of these laws in the aforementioned countries, the debate on the ethical and legal issues surrounding mandatory vaccinations remains open.

## Discussion

All sources of international law highlight that the right to health is a progressive right, whose “progressive realization” cannot be achieved in the short term. To protect this right in cases of pandemics or epidemics, it is necessary to achieve herd immunity. This is attained through widespread vaccination, which includes the pediatric population, allowing an indirect protective effect through a lower spread of infection. In fact, children develop better immunity than older adults. Vaccination of the pediatric population thereby balances the lower immunity of older adults (37). The role of herd immunity has already been demonstrated in cases of other infectious diseases, such as influenza, rotavirus, and pneumococcal disease (38–40). For this reason, vaccination of the pediatric population is important to reduce cases of MIS-C and PIMS-TS and to protect the older adults.

The COVID-19 vaccine plays a major role in protecting the population's health, reducing contagion, and accelerating the regression of the pandemic. This vaccine may become compulsory. However, compulsion is problematic in liberal democracies, which are founded on individual freedom. Jurisprudence often uses several expedients, including the fact that non-vaccination of the child will directly affect him/her, not the decision-maker. As such, compulsion might be justified in certain circumstances. In addition, extraordinary factors must be present, such as a threat to the community; the impossibility of defending a legally protected right without the restriction of freedoms (41).

Furthermore, the right to health is not the mere absence of disease, but instead includes other factors, such as those that are socioeconomic. This global model of the right to health, as the crisis between several psychophysical and socioeconomic factors, should encourage vaccination in children, since it can be a tool capable of improving their quality of life. In fact, vaccination guarantees psychological and social well-being, through the resumption of usual sociocultural activities, that were suspended during the pandemic. This suspension had some psychological repercussions, especially in the young population (42, 43). Vaccination also provides physical well-being, reducing hospital admissions and serious manifestations of infection. In fact, the authors currently support children's vaccination, believing that very often, the complications arising from highly diffusive events have a much higher incidence than the adverse effects of vaccination. Therefore, the scientific data currently available, promotes vaccination in children, since its most serious adverse effects were limited to sporadic episodes of myocarditis, with favorable prognosis. In contrast, COVID-19-related MIS-C or PIMS-TS led to severe heart and respiratory failure. In fact, although rare, the sequelae of COVID-19 infection in the pediatric population were not free from fatal outcomes (44, 45). Therefore, the risk/benefit ratio would currently be in favor of a large-scale vaccination of the pediatric population. However, in the case of “mature minors” with sufficient intelligence to understand the consequences of the proposed medical treatment, and holding the capability of discernment, the vaccine should be administered only in the presence of the will of the minor, as well as with the consent of the parents.

Furthermore, vaccination could be considered a social act equivalent to that used in a state of emergency and is necessary for the protection of the population's health. In this context, it is crucial to assess the consequences of free-riding, which endangers public health. In fact, it implies the absence of a moral duty to accept burdens, despite the benefits of a collective action (41, 46). Therefore, the COVID-19 vaccine could be considered a duty to protect public health. In this sense, vaccine-related adverse effects should be not exempt from future remediation.

Healthcare workers, for whom vaccination was mandatory, will most probably have access to compensation for vaccine-related complications. However, the judicial profile regarding vaccination compensation should be updated and expanded, including among the cause of compensation also the complications secondary to non-compulsory vaccinations, but recommended during emergencies, such as pandemics.

Further monitoring and studying late complications of the COVID-19 vaccine and infection in the pediatric population is needed. It will be possible to understand the risk/benefit ratio in the pediatric population only after obtaining more relevant scientific data. For this reason, the authors reserve the right to change their opinion if new and relevant scientific data regarding this topic emerges. Meanwhile, the bioethical debate remains open, where one wonders whether the vaccine represents a necessary tool to protect the right to health of children and the global population.

## Data Availability Statement

The raw data supporting the conclusions of this article will be made available by the authors, without undue reservation.

## Author Contributions

CB and GP conducted the researches and wrote the draft. SZ and AA oversaw and revised the draft. All authors contributed to the article and approved the submitted version.

## Conflict of Interest

The authors declare that the research was conducted in the absence of any commercial or financial relationships that could be construed as a potential conflict of interest.

## Publisher's Note

All claims expressed in this article are solely those of the authors and do not necessarily represent those of their affiliated organizations, or those of the publisher, the editors and the reviewers. Any product that may be evaluated in this article, or claim that may be made by its manufacturer, is not guaranteed or endorsed by the publisher.

## Footnotes

^1^Available online at: https://www.aopi.it/news/pazienti-pediatrici-covid-al-via-il-monitoraggio-di-aopi-il-76-dei-bambini-ricoverati-non-e-vaccinato/.

^2^Available online at: https://sip.it/2021/12/03/bambini-e-vaccino-covid-19-cosa-ce-da-sapere.

## Abbreviations

COVID-19: coronavirus disease 2019
MIS-C: multisystem inflammatory syndrome
PIMS-TS: pediatric inflammatory multisystem syndrome
SARS-CoV-2: severe acute respiratory syndrome coronavirus-2.
